# C-953-07. Nonsterile Burn Care in Practice: A Single Institution Review of Cost Savings and Infectious Outcomes

**DOI:** 10.1093/jbcr/irag033.131

**Published:** 2026-04-06

**Authors:** Piper Schneider, Raquel Arias-Camison, Mumin Sabha, Derek Bell

**Affiliations:** University of Rochester Medical Center, Rochester, NY; University of Rochester Medical Center, Rochester, NY; University of Rochester Medical Center, Rochester, NY; University of Rochester Medical Center, Rochester, NY

## Abstract

**Introduction:**

Burn wound care was historically performed using sterile personal protective equipment (PPE), though some institutions have transitioned to a nonsterile approach. Despite this shift, no consensus exists regarding the optimal standard of care. This study presents a retrospective analysis from a single institution practicing nonsterile burn care, focusing on infection rates and per-provider cost savings associated with nonsterile PPE use.

**Methods:**

Data were collected from January 2015 to May 2025 on burn-related infection complications, length of stay, and operative timelines for surgical (n = 1006) and non-surgical (n = 906) admitted, inpatient burn patients. Outpatient procedures were excluded. PPE costs were calculated using institutional supply prices. Sterile PPE included one bouffant, one pair of sterile gloves, one sterile gown, and one surgical mask. Nonsterile PPE included one pair of non-sterile gloves. All patients received daily dressing changes. Dressing change totals were calculated using hospital length of stay for non-surgical patients and days prior to operation for surgical patients.

**Results:**

Over the 10-year period, 7 years of data could be extracted with 15 707 nonsterile dressing changes performed and an infection rate of 0.7% per patient and 1.1 infections per 1000 dressing changes. Average TBSA was 5.3 ± 9.3% with 78 010 open wound days. The per-provider cost of sterile PPE was $3.77, compared to $0.12 for nonsterile PPE, resulting in a savings of $3.65 per dressing change. This equated to a total institutional savings of $57330.55 per provider over 7 years. Estimating 3 providers per dressing change yields $171991.65 total savings.

**Conclusions:**

This single-institution experience demonstrates that nonsterile burn care can maintain low infection rates while significantly reducing PPE costs.

**Applicability of Research to Practice:**

These findings support the broader adoption of nonsterile burn care protocols as a safe, cost-effective alternative in appropriate clinical settings.

**Funding for the study:**

N/A.

